# Evaluation of phylogenetic reconstruction methods using bacterial whole genomes: a simulation based study

**DOI:** 10.12688/wellcomeopenres.14265.2

**Published:** 2018-05-29

**Authors:** John A. Lees, Michelle Kendall, Julian Parkhill, Caroline Colijn, Stephen D. Bentley, Simon R. Harris

**Affiliations:** 1 Infection Genomics, Wellcome Sanger Institute, Hinxton, Cambridgeshire, CB10 1SA, UK; 2 Department of Microbiology, New York School of Medicine, New York, 10016, USA; 3 Department of Mathematics, Imperial College London, London, SW7 2AZ, UK

**Keywords:** phylogeny, simulation, tree distance, bacteria, phylogenetic methods

## Abstract

**Background**: Phylogenetic reconstruction is a necessary first step in many analyses which use whole genome sequence data from bacterial populations. There are many available methods to infer phylogenies, and these have various advantages and disadvantages, but few unbiased comparisons of the range of approaches have been made.

**Methods**: We simulated data from a defined 'true tree' using a realistic evolutionary model. We  built phylogenies from this data using a range of methods, and compared reconstructed trees to the true tree using two measures, noting the computational time needed for different phylogenetic reconstructions. We also used real data from *Streptococcus pneumoniae* alignments to compare individual core gene trees to a core genome tree.

**Results**: We found that, as expected, maximum likelihood trees from good quality alignments were the most accurate, but also the most computationally intensive. Using less accurate phylogenetic reconstruction methods, we were able to obtain results of comparable accuracy; we found that approximate results can rapidly be obtained using genetic distance based methods. In real data we found that highly conserved core genes, such as those involved in translation, gave an inaccurate tree topology, whereas genes involved in recombination events gave inaccurate branch lengths. We also show a tree-of-trees, relating the results of different phylogenetic reconstructions to each other.

**Conclusions**: We recommend three approaches, depending on requirements for accuracy and computational time. For the most accurate tree, use of either RAxML or IQ-TREE with an alignment of variable sites produced by mapping to a reference genome is best. Quicker approaches that do not perform full maximum likelihood optimisation may be useful for many analyses requiring a phylogeny, as generating a high quality input alignment is likely to be the major limiting factor of accurate tree topology.  We have publicly released our simulated data and code to enable further comparisons.

## Introduction

Phylogenetic analysis is a complex task, but one that is foundational to many applications in bacterial genetics: molecular evolution, outbreak tracing and genomic epidemiology, to name a few ^1, 2^. The modern genomic analyst faces a bewildering array of options at every stage of the process.

The possible number of trees for even a small number of tips is enormous ^3^ – for 96 tips there are 10 ^173^ possible trees (compare this to 10 ^80^ atoms in the observable Universe, or even 10 ^120^ possible games of chess). Fortunately, sophisticated software methods allow us to sensibly navigate through this space to the most likely trees.

Generally the steps taken when analysing a population of bacteria that have been whole genome sequenced are as follows. Quality control of the raw data must first be performed, after which a whole-genome alignment of the sequences is produced. The alignment is usually produced by mapping reads to a reference sequence (of which many likely exist), but may also be obtained by *de novo* assembly followed by whole-genome alignment (either by progressive local alignment, or through multiple sequence alignment of orthologous genes and intergenic regions). Many methods are available to map reads to a reference, assemble reads into contigs and align contigs or genes, and each method will typically have many options. This alignment is the key input for phylogenetic inference software. Even more methods, with yet more complex options, exist to determine the most likely phylogeny given a sequence alignment. Alternatively, one may forgo alignment altogether, and opt instead for a k-mer distance-based approach followed by a neighbor joining tree.

Understandably, this complexity and range of choice means that methods sections of papers using phylogenetic analysis are often different between studies. This disparity is likely due to different software preferences (familiarity, speed and usability being major factors in this choice), rather than an informed choice based on the biological question and resources to hand. One should carefully consider what question the tree is trying to address: is it to look at overall population structure, or to try and find precise relationships between closely related isolates? The relative merits of different approaches are difficult to objectively assess, even after careful reading of the original method manuscripts. The potential effect of different combinations of approaches at each step in the process between raw sequence reads and the final phylogeny has seldom been explored.

It is therefore desirable to provide a comparison between phylogenetic methods that is focused on methods’ ability to answer the biological question at hand. Some previous attempts have been made, using either simulated data, experimental evolution, or an assumption that the maximum likelihood phylogeny is correct. One such study assessed the running times and likelihood of trees drawn from simulated data using two pieces of software ( RAxML and FastTree), assuming the model of sequence evolution is correct ^4^. A larger study in eukaryotes compared these two methods with IQ-TREE in terms of the best likelihood obtained using both species and gene trees ^5^. Other small-scale comparisons include a comparison of read-to-tree pipelines with other pieces of software ^6^, and the production of “well characterised” reference datasets for testing methods ^7^. A recent study instead used an *Escherichia coli* hypermutator to conduct experimental evolution along a defined balanced phylogeny, and then by sequencing the strains at the tips, the authors compared the ability of 12 combinations of methods to reconstruct the correct phylogenetic relationship ^8^. An overview of how the most commonly used combinations of methods perform in terms of phylogeny accuracy, as opposed to best likelihood, does not yet exist. Comparison of likelihoods alone assumes that we know the true evolutionary model, and doesn’t allow us to evaluate in what way the tree is wrong.

In this paper we present a simulation-based analysis of the speed, ease of use, and accuracy of some of the common ways to obtain a phylogeny from bacterial whole genome sequence data. We define a true tree, from which we produce whole genome sequence data using realistic simulations (thereby avoiding the problem of circularity of model choice). A range of methods are then evaluated for accuracy using appropriate metrics in tree space. We hope to provide some insight into which approaches should be favoured in certain settings while acknowledging that our simulations are far from comprehensive. We also make our code and simulated data publicly available in the hope that this might inspire further method comparisons aimed at different settings.

## Methods

### Simulating bacterial populations – assemblies and alignments

We wished to simulate genomes in a realistic way, without using the same model of evolution that any one software package uses to compute tree likelihoods or sequence distances in order to reconstruct the tree. This would be circular, and would result in that software package necessarily performing best.

**Figure 1.**
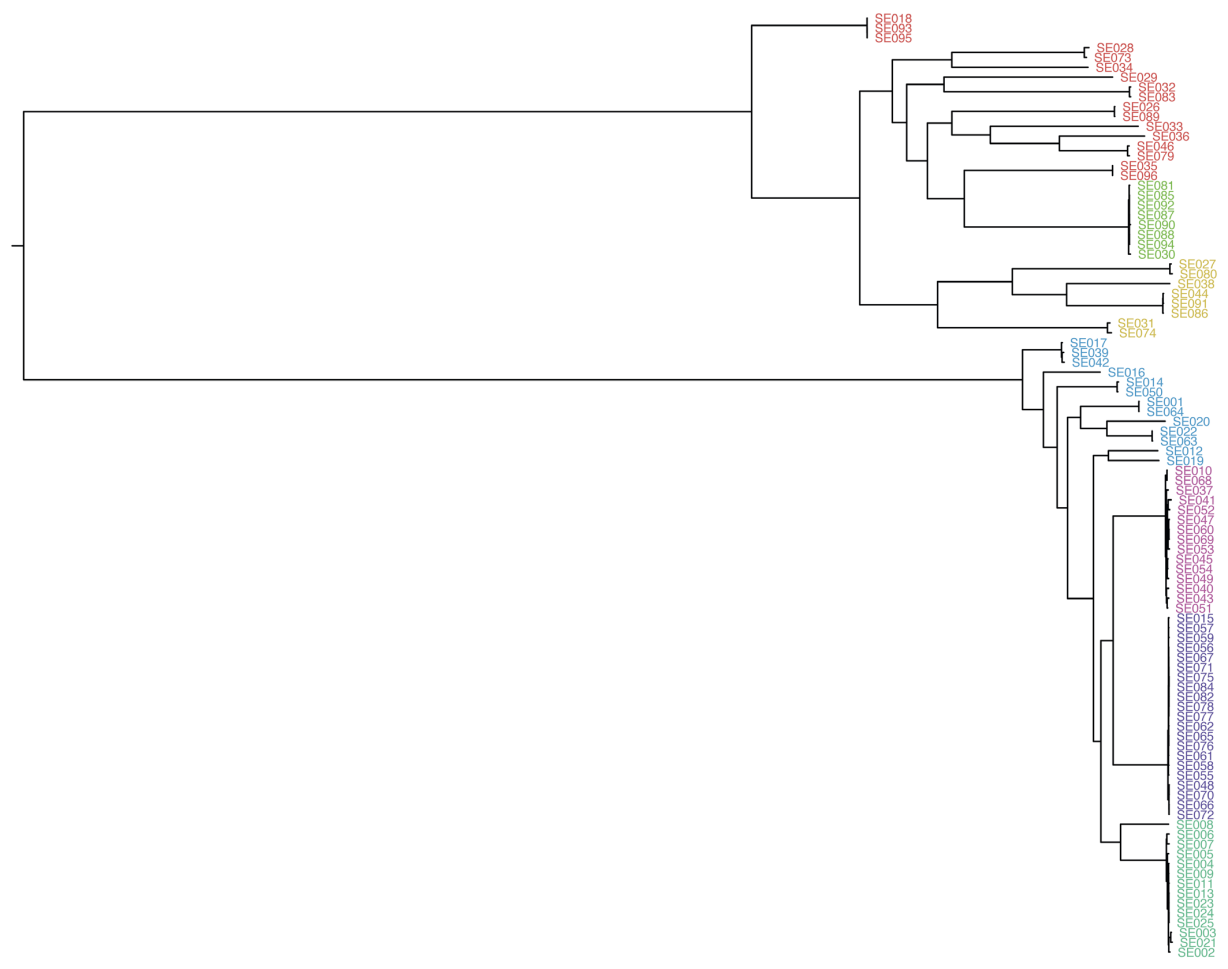
The phylogeny inferred by Kremer *et al.* ^12^ used as the true tree in simulations. Tips are coloured by BAPS cluster inferred from the core genome alignment.

For the simulations we used parameters for *Streptococcus pneumoniae*, whose evolution has been extensively studied using genomic data, but artificially used a tree topology from another species which had desirable properties for downstream comparisons. We therefore used Artificial Life Framework v1.0 (ALF) ^9^ to simulate evolution along a given phylogenetic tree, for the 2 232 coding sequences in the *S. pneumoniae* ATCC 700669 genome ^10^ as the MRCA. As well as modeling SNP evolution, ALF also allows for short insertions and deletions (INDELs), gene loss and horizontal gene transfer events which occur in real populations but are usually not included in phylogenetic models. In parallel, we used DAWG v1.2 ^11^ to simulate evolution of intergenic regions (defined as sequence not annotated as a CDS). We identified a phylogeny ( Figure 1), originally produced by Kremer *et al.* ^12^ from a core genome alignment of 96 *Listeria monocytogenes* genomes from patients with bacterial meningitis which possessed a number of qualities we wished to be able to reproduce. Particularly, it had two distinct lineages (also making midpoint rooting suitable, and negating the strong dependence on correct rooting implicit in the Kendall and Colijn metric ^13^), several clonal groups within each lineage, long branches and a polyphyletic population cluster. Population clusters were estimated from the resulting core genome alignment from simulations using Bayesian Analysis of Population Structure v6.0 (BAPS) ^14^. We define *N* as the number of strains in the study and *M* as the number of aligned sites.

We used realistic parameters, as far as possible, for the simulation run with ALF. To estimate rates to use in the generalised time-reversible (GTR) matrix and the size distribution of INDELs, we first aligned *S. pneumoniae* strains R6 (AE007317), 19F (CP000921) and *Streptococcus mitis* B6 (FN568063) using Progressive Cactus v0.0 ^15^. This whole genome alignment allowed calculation of SNP and INDEL rates for these models. We used previously determined parameters for the rate of codon evolution ^16^, relative rate of SNPs to indels in coding regions ^17^, rates of gene loss and horizontal gene transfer ^18^ when running the simulation. We then used ALF with these parameters to simulate the evolution of coding sequences from the root genome along the given phylogeny. For the intergenic regions we used the same GTR matrix parameters and previously estimated intergenic SNP to INDEL rate ^17^. We combined the resulting sequences of coding and non-coding regions at tips of the phylogeny while accounting for gene loss and transfer, and finally generated error prone Illumina reads from these sequences using pIRS v1.11 ^19^. An overview of this process is shown in Supplementary Figure 1 ( Supplementary file 1).

To generate input to phylogenetic inference algorithms, we created assemblies and alignments from the simulated reads. We assembled the simulated reads into contigs with velvet v1.2.09 ^20^ using https://github.com/tseemann/VelvetOptimiser to choose an optimal coverage cutoff and k-mer size (between 37 and 81). We then improved and annotated the resulting scaffolds using the sanger-pathogens improvement pipeline with default parameters ^21^. We generated alignments by mapping reads to the TIGR4 reference using bwa-mem v0.7.10 with default settings ^22^, and called variants from these alignments using samtools v1.2 mpileup and bcftools call ^23^. We used Roary 1.007001 ^24^ with a 95% BLAST ID cutoff to construct a pan-genome from the annotated assemblies, from which a core gene alignment was created with MAFFT v7.205 ^25^. Downstream analysis using genes was done using this pan-genome. We then created alignments using two further methods. For an MLST-like alignment we selected seven genes at random from the core alignment (present in all strains) which had not been involved in horizontal transfer events. For a Progressive Cactus alignment, we ran the software on the assemblies using default settings, and extracted regions aligned between all genomes from the hierarchical alignment file and concatenated them.

### Methods of phylogeny reconstruction

Using the nucleotide alignments described above as input, we ran the following phylogenetic inference methods:

- RAxML v7.8.6 ^26^ with a GTR+gamma model (-m GTRGAMMA).
- RAxML v7.8.6 with a binary+gamma sites model (-m BINGAMMA).
- IQ-TREE v1.6.beta4 ^27^ using a GTR+gamma model (-m GTR+G) (denoted slow) and using GTR and the -fast option (denoted fast).
- IQ-TREE v1.6.beta4 with mixed partitions with matched branch lengths and varying evolutionary rates (-spp). We used a GTR+gamma model (-m GTR+G) for the SNP alignment, and a binary GTR model (-m GTR2) for gene presence/absence.
- FastTree v2.1.9 ^28^ using the GTR model (denoted slow) and using the -pseudo and -fastest options (denoted fast).
- Parsnp v1.2 ^29^ on all assemblies using the -c and -x options (removing recombination with PhiPack).

We attempted to run the REALPHY v1.12 pipeline ^6^, but it was not computationally feasible due to the slow mapping step (using bowtie2) not being parallelisable by strain.

We also created pairwise distance matrices using:

- Mash v1.0 ^30^ (default settings) between assemblies.
- Andi v0.9.2 ^31^ (default settings) between assemblies.
- Hamming distance between informative k-mers using a subsample of 1% of counted k-mers from assemblies ^32^.
- Hamming distance between SNP sites produced by Disty McMatrixface v0.1.0.
- JC and logdet distances between sequences in the alignment, as implemented in SeaView v4.0 ^33^.
- Distances between core gene alleles (present in 100% of isolates) from the roary alignment. We added a distance of zero for each core gene with identical sequence, or added a distance of one if nonidentical, as used in the BIGSdb genome comparator module ^34^.
- Normalised compression distance (NCD) ^35^, using PPMZ as the compression tool ^36^.

For all the above distance matrix methods we then constructed a neighbor joining (NJ) tree, a BIONJ tree ^37^ using the R package ape, and an UPGMA tree using the R package phangorn. In the comparison we retained the tree building method from these three with the lowest distance from the true tree (see below).

### Quantifying differences between phylogenetic tree topologies

To measure the differences in topology between the produced trees (either between the true tree and an inferred tree, or between all different inferred trees) we used two measures. As a sensitive measure of changes in topology we used the metric proposed by Kendall and Colijn ^13^ setting *λ* = 0 (ignoring branch length differences). We choose to ignore branch length differences as maximum likelihood methods (which will perform much better) will not be comparable with distance based approaches. We also decided that topology difference was more intuitive over the range of methods we tried, rather than the combination of topology and branch lengths that setting *λ >* 0 would give. We compared the true tree against randomly generated trees from the ape function rmtree, which randomly splits edges. After midpoint rooting this gave 286 (95% CI 276–293) as a comparison to poor topology inference. To illustrate how these numbers correspond to actual changes in topology we used the *plotTreeDiff* function from the treespace package for three representative comparisons (see interactive treespace plots or static Supplementary Figure 2– Supplementary Figure 5 ( Supplementary File 1).

For trees distant from the true tree by the KC metric it was useful to test whether the tree was accurate overall and only a few clade structures were poorly resolved, or whether the tree failed to capture important clusters at all. We therefore checked the clustering of the BAPS clusters from the true alignment on each inferred tree. We did this with both the primary BAPS cluster, which separates the two main lineages, and the secondary BAPS clusters which define finer structure in the data and includes a polyphyletic cluster. For each BAPS cluster, we assessed whether tips were clustered correctly by checking whether it was still monophyletic in the inferred tree, and whether the polyphyletic cluster was still split in the same way.

### Core gene trees from real data

We used a previously generated core genome alignment from 616 *S. pneumoniae* samples isolated from the nasopharynx of asymptomatically carrying children in Massachusetts ^38– 41^. We ran IQ-TREE on the whole alignment using a GTR model (-m GTR). We then aligned each core gene at the codon level with RevTrans v1.10 ^42^, and then ran IQ-TREE on each nucleotide alignment using the same model. We calculated the KC metric with *λ* = 0 between all these pairs of trees, and used treespace to perform multi-dimensional scaling in two dimensions to visualise the pair-wise distances ^43– 45^.

## Results

**Figure 2.**
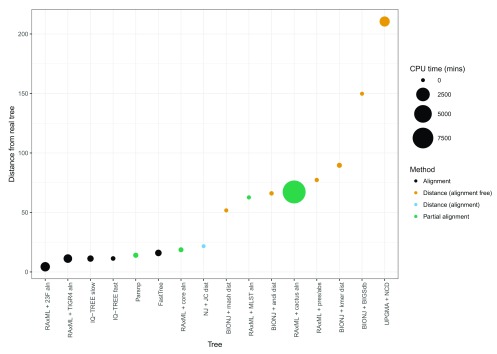
Ordered accuracies from Table 1, showing the CPU time required for each tree. There are large changes in accuracy between the alignment and distance methods, and again between two inaccurate distance methods.

Table 1 and Figure 2 show the results of our simulations, ranked by their KC distance from the true tree. We note that all methods except for the NCD were able to recapitulate the population clusters as defined by BAPS. Additionally, all methods found a consistent midpoint root. This is reflected by the KC metric scores which would be significantly higher if there were ‘deeper’ differences in the tree topologies, particularly concerning the root position.

**Table 1.** Accuracy and resource usage of phylogenetic reconstruction methods, ordered by KC metric score. The method lists the best combinations of all alignment with phylogenetic method, and distance matrices with phylogenetic methods. Three scores of accuracy of the phylogeny are shown; the KC metric is described in the text, the BAPS scores (the primary and secondary clusters, respectively) are a tick if the clusters are as in the true tree, otherwise which clusters are wrong (all clusters, or just the polyphyletic clusters). Parallelisability shown is that built into the software, “completely” is when every value in a distance matrix is independent so can be parallelised up to *N* ^2^ times. Accessory indicates whether accessory elements (not present in all isolates) are used in the phylogenetic inference.

Method	KC (0-286)	BAPS 1	BAPS 2	CPU time	Memory	Overheads	Parallelisability	Accessory genome?	Recommended
RAxML + close reference alignment	4.63	✓	✓	806.5 minutes	2.7 Gb	Mapped alignment	Pthreads	No	NA (artificial)
RAxML + alignment	11.2	✓	✓	587 minutes	3.0 Gb	Mapped alignment	Pthreads	No	Accurate but slow
IQ-TREE (slow) + alignment	11.2	✓	✓	703 minutes	3.2 Gb	Mapped alignment	Pthreads or MPI	No	Accurate but slow
IQ-TREE (fast) + alignment	11.3	✓	✓	14.6 minutes	1.1 Gb	Mapped alignment	Pthreads or MPI	No	Accurate/fast tradeoff
Parsnp	14.0	✓	✓	42.5 minutes	2.6 Gb	Assemblies	Threads	No	Artificial
FastTree + alignment	16.0	✓	✓	189 minutes	10.6 Gb	Mapped alignment	Threads (up to 4)	No	Accurate/fast tradeoff
RAxML + core gene alignment	18.6	✓	✓	29.2 minutes	154 Mb	Core gene alignment	Pthreads	No	Comparable to mapping
NJ + SNPs alignment	20.5	✓	✓	Negligible	Negligible	Mapped alignment	No	No	No
IQ-TREE + mixed partitions	24.5	✓	✓	1316 minutes	3.2Gb	Mapped alignment + accessory genes	Pthreads or MPI	Yes	No
BIONJ + mash distances	51.7	✓	✓	0.75 minutes	10 Mb	Assembly	Completely	Yes	Best, when no alignment
RAxML + Seven gene alignment (MLST- like)	62.6	✓	✓	1.4 minutes	19 Mb	Assembly	Pthreads	No	No
BIONJ + andi distances	66.0	✓	polyphyly	7.48 minutes	290 Mb	Assembly	Completely	Yes	No
RAxML + Cactus alignment	67.2	✓	✓	9 600 minutes	37.4 Gb	Assembly	Threads	No	No
RAxML + gene presence/absence	77.3	✓	polyphyly	4.28 minutes	20 Mb	Core gene alignment	Threads	Yes	No
BIONJ + k-mer distances	89.6	✓	✓	37.3 minutes	180 Mb	Assembly	Threads	Yes	No
NJ + ANI/ Hamming distances	98.1	✓	polyphyly	Negligible	230 Mb	Mapped alignment	No	No	No
BIONJ + BIGSdb- like	150	✓	polyphyly	0.48 minutes	Negligible	Assembly	Completely	No	No
UPGMA + NCD	210	✓	all	1 040 minutes	Negligible	Assembly	Completely	Yes	No

For construction of a maximum likelihood (ML) tree, RAxML is one of the most heavily used and efficient software methods available. As expected, this was the most accurate method tested, and also the most resource heavy (apart from whole-genome alignment, discussed later). RAxML’s model is a close fit to the model used to generate the data, and this model is expected to be a good model of evolution. There was no significant difference in the likelihood of the fit of the inferred tree and the true tree under this model (LRT = 2.34; p = 0.13). When using an alignment against a different reference genome from the one we actually used in the simulations, as is more likely to be the case in real alignment production, RAxML was tied for accuracy with IQ-TREE which also produced the same tree. In our simulations RAxML had better resource requirements than IQ-TREE, though over a range of data the programs are likely comparable.

A common consideration with ML trees from alignments is whether to include all sites, or remove the constant sites and analyse just SNP sites. The potential advantage of the latter approach is to reduce memory usage, which is particularly important when analysing huge alignments with thousands of sequences. Selecting just the polymorphic sites introduces an ascertainment bias which can cause branch lengths to be overestimated, so a correction needs to be applied to prevent this ^46^. Both RAxML and IQ-TREE implement this correction, so we compared tree accuracy and resource use between these two modes ( Supplementary Table 1; Supplementary file 1). We found similar topology in both modes, and if anything more accurate branch lengths when using polymorphic sites with an ascertainment bias correction. Most importantly, resource use (CPU time and maximum memory use) was much lower when using only variable sites – we would therefore recommend this approach over using the full alignment.

### Partial alignment methods or alternative reconstruction give good trees

Knowing the quality of maximum likelihood trees, one approach a user may take to reduce the large computational requirements is to reduce the number of sites *M* that are included in the alignment. Some common ways this can be achieved are either by finding clusters of orthologous genes and only using sites from “core” genes (those present in every sample), or by using an alignment of the pre-defined MLST genes. In this test we found that using a core genome alignment slightly reduced the accuracy, whereas using an alignment of seven genes, similar to MLST, reduced the accuracy greatly, as only a small proportion of the genomic variants are now used in the inference.

Other than as a way to reduce computational burden, core genome alignment may increase the accuracy of the input alignment by excluding mismapping of repetitive regions and minimising bias from missing data in accessory genes. However, there is the issue that when a variant is present in a region overlapped by two genes it will be erroneously represented twice. When analysing a whole species, particularly when the core genome contains only a fraction of the overall diversity, this can also lead to a loss of resolution within lineages. One way to avoid this is by first defining lineages, then producing a separate alignment and tree for each. In this case one should take advantage of multiple reference genomes by selecting one that is genetically close to each lineage to produce the alignment.

When performing phylogenetic analysis, the user should consider whether they want to include the accessory genome in their inference (final column in Table 1). In this simulation, evolution of the core and accessory genome are correlated, so that including the accessory genome improves accuracy over using core genome alone. In a species such as *Streptococcus pneumoniae* where multiple distinct lineages are maintained over time, the core and accessory evolution tend to be correlated in this way ^47^. In some other species, for example *Staphylococcus aureus* ^48^, the accessory genome is dominated by mobile elements such as transposons and phage (the same is also true within a single lineage of *S. pneumoniae*). In species such as *Escherichia coli* accessory genes are highly mobile ^49^. In both cases the evolutionary signal from accessory genes is discordant from core genome evolution, so including these in the alignment will not give a good estimate of vertical evolutionary distance between strains. In other situations the core and accessory genome may both carry signals of vertical evolution, but they may be discordant with each other due to different evolutionary processes acting on each type of variation. A binary model of evolution can be used to build a maximum likelihood tree based on accessory gene gain and loss (RAxML + gene presence/absence), but we found that its accuracy is much lower than a model of SNP variation within genes. A possibility for combining these two data types would be to have separate model partitions for SNP variation and gene gain/loss. We have provided an example of this using IQ-tree on the simulated data, though we found this actually reduced accuracy of the resulting topology (KC score 24.5). Possible issues with this approach are that genes which are discordant with the phylogenetic signal from vertical evolution of the core genome (e.g. mobile genetic elements) may reduce accuracy, and incorrectly split orthologues in the accessory genome.

**Figure 3.**
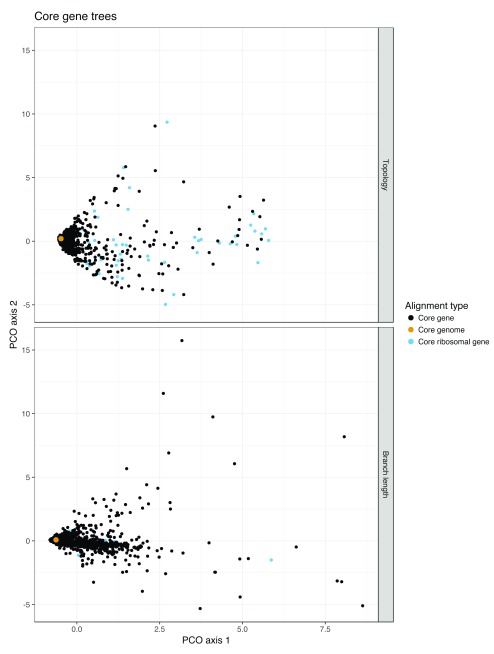
A multidimensional scaling plot of the KC distances between all core gene trees from a real population of 616 *S. pneumoniae* genomes. Top: topology distances ( *λ* = 0); bottom: branch length distances ( *λ* = 0). The core genome tree from the concatenated alignment is shown in yellow; trees from ribosomal proteins, which tended to have different topologies due to their lack of variation, are shown in blue. The top twenty divergent trees by branch length are listed in Supplementary Table 2 ( Supplementary File 1). The full list of distances by gene can be accessed at https://gist.github.com/johnlees/da164a4260e13528e8315e266a46bf3f.

To further investigate core genome alignment, we compared individual gene trees to a core genome tree in a real population of *S. pneumoniae* genomes. We created trees from all core genes, and compared them by projecting pairwise KC distances into two dimensions ( Figure 3). The figure shows that the core genome tree behaves like an ‘average’ of the individual core gene tree topologies, without being biased by the bad topologies produced at distances far from the center of the main cluster. Looking at the distant topologies, we found that the genes giving these trees were mostly ribosomal related proteins. These alignments contained very little variation due to their highly conserved function, providing little information for phylogenetic resolution – the root and ancestral part of these topologies were different from the core genome alignment tree, likely due to random placement of nodes, giving highly divergent KC distances. Reassuringly, concatenating these 82 ribosomal gene alignments and producing a tree performed better than any individual gene alignment (KC distance = 1362), giving more confidence in rMLST schemes.

The gene trees closest to the whole core gene alignment tree were those with the most variation. When we included branch lengths in the distance measure ( *λ* = 1 in the KC metric), very short branch lengths contribute far less to the tree distance than longer lengths, and the ribosomal genes are no longer outliers. Many of the furthest gene trees from the core genome tree are from genes known to be involved in recombination events ^50^, as shown in Supplementary Table 2 ( Supplementary File 1). Recombinations result in a large number of SNPs against a reference; because phylogenetic methods assume vertical evolution, recombination tends to inflate estimated branch lengths, but generally do not affect topology ^51^. The best practice is to try to remove these regions before performing phylogenetic reconstruction ^52^. When picking an MLST scheme for an organism the most important considerations are probably recapitulation of epidemiological parameters, ease and consistency of use ^53^. However, given a choice of suitable genes to use, ranking of these phylogenetic signals may be a useful additional consideration. Searching through combinations of different gene alignments suggested little interaction between them affecting the final topology; the upshot being that genes that individually perform well can be considered as candidates without worrying about the specific combination chosen.

We also evaluated the quality of a phylogeny drawn from a progressiveCactus alignment ^15^, which performed best in a comparison between whole genome aligners ^54^. Whole genome alignment uses linear sequences in an annotation-free manner, and by breaking the alignment job into smaller local regions can align sequences in the presence of structural variation such as gene gain and loss, inversions and transversions – both core and accessory elements are aligned. In this comparison, the core genome alignment we extracted was smaller than that produced by Roary, and therefore produced a less accurate phylogeny. This class of methods is therefore best suited to comparing small numbers of genomes from larger evolutionary distances (across species), rather than large numbers of more closely related genomes.

In the search for greater computational efficiency, rather than changing the alignment one may instead opt to use a different method of phylogenetic inference. One piece of software which aims to infer phylogeny faster than a maximum likelihood method, albeit at the expense of accuracy, is FastTree ^28^. In our test FastTree ran four times faster than RAxML, without much decrease in accuracy. We found little difference in accuracy when using the fast and slow options. The scaling of CPU time in FastTree by number of sequences is more favourable than RAxML, so as the number of sequences increases the relative speedup of FastTree will also increase. It should also be noted that FastTree obtains around a 2x speedup from using four CPUs using OpenMP, whereas RAxML can use around 16 threads at close to 100% efficiency.

Parsnp ^29^ produces a core genome alignment by rapidly finding maximal exact matches (MEMs, as in nucmer) which can include both genes and intergenic regions. The use of MEMs means that assembly quality will affect parsnp results, which was designed for use with reference-quality genomes (for example, those produced by SMRT sequencing. In our test we found that it performed even better than FastTree while using less CPU time, however our assemblies from simulation are likely more amenable to comparison of MEMs than real data, which is more fragmented. The method does not deal well with mobile elements or recombination, so extra caution should be used with real datasets where this variation is prevalent.

Finally, we saw very promising results when using the “fast” mode of IQ-TREE, currently available in beta. Reconstruction in this case was as accurate as a full maximum likelihood method, and completed quickly with modest memory requirements. Once available as a stable release, this may prove to be the most accurate way to efficiently infer large phylogenies.

### Genetic distance based approaches rapidly give a rough tree topology

Early phylogenetic methods involved drawing a neighbour joining tree from a matrix of pairwise distances between all tips. This method is fast and simple. When we used distances calculated from the same alignment as RAxML this approach was somewhat worse than the reduced number of sites or reduced accuracy methods above, but still gave a good overall topology – better than an ML tree from seven core genes (similar to MLST). A tree can also be drawn from distances using BIONJ, which by using a simple evolutionary model can be expected to provide trees with more accurate topologies than NJ ^37^. Another alternative is UPGMA, though as a hierarchical clustering method it would not be expected to recover the same topology as a phylogenetic method (but perhaps the same clusters).

However, in the present era, we see the main advantage of this class of methods as being able to avoid having to create an alignment from mapping ^55^. If one is able to calculate genetic distances from assemblies or even directly from reads, the relatively costly and challenging step of creating a large multiple sequence alignment can be avoided. Although *N* ^2^ distances need to be evaluated, these calculations are independent so the process is trivially parallelisable. We tried creating trees from five methods which can evaluate pairwise distances rapidly: mash, andi, k-mer distances, BIGSdb and the normalised compression distance (NCD).

The NCD is a general method to compare the similarity between any two data objects ^35^. The NCD between two objects *x* and *y* (in this case the sequence of assemblies) is computed as follows:

$$\text{NCD}\left(x,\;y\right)=\frac{Z\left(x,\;y\right)-\min\left[Z\left(x\right),\;Z\left(y\right)\right]}{\max\left[Z\left(x\right),\;Z\left(y\right)\right]}$$

where *Z*( *x*) is the size after compression of file *x*. The rationale is that the more two sequences are similar to each other, then the more the compression method will be able to use this similarity to reduce the overall size of the concatenated file towards the lower limit of the size of the compressed individual files. We used PPMZ as the compressor to avoid issues with minimum block size ^36^, but only recovered the largest scale feature of the two main lineages in the topology. This suggests the the NCD is not well suited to finding distances between sets of closely related sequences, but may perform better with more distant genomes. PPMZ may not be the best compressor overall due to its long run time, but we did not investigate this further.

BIGSdb is a database designed to store bacterial sequences, and perform pre-defined analysis rapidly on them ^34^. Trees from genomes in this database can be produced with the GenomeComparator module. This works by comparing the alleles of core gene sequences, increasing the distance between two genomes by one for each allelic difference between the genes that they have. The potential advantage of this is that recombination events will correctly be counted as a single evolutionary change, rather than as multiple separate SNP differences. However, this approach also limits resolution and inference of intra-cluster distances, and produced one of the worst topologies in our tests.

Finally, we used k-mer distances ^32^, mash ^30^ and andi ^31^ to create distance matrices. andi counts the number of mismatches between equally spaced maximal exact matches between a pair of sequences. mash was partly designed as an improvement to the accuracy of andi, and instead uses the MinHash algorithm to rapidly approximate the Jaccard distance between the sets of k-mers in each assembly. This is also the distance approximated by our k-mer method, but is many-fold more efficient due to the use of MinHash. In our test, we found that mash performed the best out of any distance-based measure in accuracy and efficiency, but was still significantly less accurate than the alignment-based methods. Considering the ease of use and efficiency of mash, its ability to recover population clusters means that it could be recommended as the tool of choice for first-pass analysis.

## Discussion

**Figure 4.**
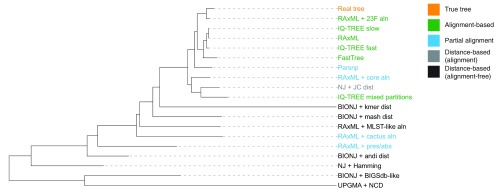
Tree of tree methods. Using the KC metric between all the inferred phylogenies in Table 1 to create a pairwise distance matrix, an NJ tree created from this matrix. This shows how the topologies from all methods are related to each other (a tree-of-trees, or supertree). The true tree is in orange at the top, and four classes of methods are labeled. For alignment-based methods the mapping of reads to the TIGR4 reference was used, unless explicitly stated. We also performed multi-dimensional scaling of these distances in two dimensions to show how the methods clustered (see interactive treespace plots or static Supplementary Figure 6; Supplementary File 1).

We have analysed the ability of a range of phylogenetic inference methods to reproduce the topology and clustering of a known tree when given realistic simulated data derived from the same known tree. Figure 4 shows an alternative presentation of our results: a tree-of-trees, also showing the ways in which some of the incorrect trees may be similar to each other.

Overall, we found that modern maximum likelihood methods and a good alignment can obtain an accurate phylogeny in reasonable runtimes; using approximate phylogeny methods with a good alignment is the next best thing, followed by reducing the alignment size. The best quality results had the longest computational time requirements, consistent with our mechanistic understanding of how phylogenetic inference should perform. We would expect maximum likelihood approaches to do well on molecular data, and to take more time than distance based methods ^56^. For rough analysis, genetic distances as produced by mash can be used for clustering and to produce a rough coarse-grained topology. Consideration of whether to include the accessory genome in the inference or to analyse it separately is important, and will be dependent on the species and lineage being studied.

Choice of method will also depend on why the tree is being built in the first place. If it is for overall population structure, then a more approximate approach will likely suffice, as such analysis is unlikely to delve into precise topology differences at the tips of the tree. All the approaches we recommend were able to recover the correct population clusters with the simulated data. However, for purposes such as transmission cluster inference or association of epidemiological traits (for example a switch in location of isolation) a more precise topology may then be desired.

We also directly compared a range of evolutionary models, run both using BIONJ and ML ( Supplementary Table 3; Supplementary File 1). As there are a huge number of sites, and the sites are each low-dimensional, we are much better informed about the site evolution model than the tree. It’s easier to get the tree wrong, and hence the inference method used is a more important consideration for tree accuracy. We do note that simpler evolutionary models require less CPU time to run for comparable accuracy. Although maximum likelihood methods cope with missing data much better than distance methods, the extensive missing calls in these simulations (20–40% of sites, due to accessory genes) did not prevent the distance based methods from giving an approximate topology.

For a small number of samples or if computational resources are not a concern, and for phylogenetically focused questions such as model comparison, then a maximum likelihood method is the best choice. However a key point is that in many cases, especially when using a large number of genomes and especially across species with little phylogenetic signal, the phylogeny building software is not the limiting factor in accuracy of the resulting tree. The alignment used is crucial: the quality of sequencing and mapping, whether mobile elements have been masked, and how much confounding signal from recombination and homoplasy can be removed all have important effects on the quality of the final tree. In many cases the observed data are not consistent with a single phylogenetic tree, so rather than aiming for the “best” tree it is important to assess uncertainty in the tree. Bayesian methods are available but are slow and complex ^57, 58^ – we show an example of these on our simulated data in Supplementary Figure 7 ( Supplementary File 1). In many cases we would therefore recommend using a faster method such as IQ-TREE’s fast mode or FastTree, combined with bootstrap analysis to more efficiently estimate the uncertainty in tree topology ^59^. We do note that the bootstrap estimate may be difficult to interpret, as it does not behave as a standard confidence interval due to the implicit assumption that sites are independent ^60^. A recent update to the bootstrap may instead be easier to interpret ^61^, or using the KC metric to compare bootstrap trees ^62^.

For truly enormous datasets, particularly in cases where producing an alignment is the limiting step, even these approximate methods may prove intractable. In which case using pairwise distances from mash is an alternative approach. One possible problem with mash is that closely related sequences can have a distance of zero, but this can be solved by increasing the sketch size with little extra computational burden. We also note that though the MinHash distance is an approximation, it is a good one, and unlikely to be the limiting factor in these analyses. Instead, accessory genome and mobile elements may be a problem. In these simulations we also tested mash using the core alignment directly, but this resulted in a less accurate tree (KC distance = 71.6); the k-mers sampled by mash do not utilise the information of homology implicit in each column of the alignment.

This work is of course somewhat limited in initial scope. While we tried to choose a true tree with common features, the simulations here are limited, with parameters chosen to model a single species. We also made the choice to ignore branch length differences (though these can as easily be compared) as we think that topological distance is more intuitive, especially for larger differences.

In an age of a bewildering array of options for this analysis and few available direct comparisons we hope that our results are nonetheless instructive, and that these methods can continue to be compared using other benchmark datasets as they appear.

## Data availability

Data can be downloaded from the following URLs:

- Code: https://github.com/johnlees/which_tree (GPLv2 license)
- Distances of real gene trees: https://gist.github.com/johnlees/da164a4260e13528e8315e266a46bf3f
- Inferred trees: https://dx.doi.org/10.6084/m9.figshare.5483464 ^63^
- Interactive treespace plots: https://dx.doi.org/10.6084/m9.figshare.5923300 ^64^
- Simulation parameters and results (including true alignments of all genes, assemblies and annotations from simulated reads): https://dx.doi.org/10.6084/m9.figshare.5483461 ^65^

## Supplementary material

**Supplementary File 1 - File contain the following supplementary tables and figures:**

**Supplementary Table 1**: Comparison of phylogeny accuracy using all positions versus SNPs plus an ascertainment bias correction for maximum likelihood methods. The KC distance from the true tree, using topology only ( *λ* = 0) and including branch lengths ( *λ* = 1) is shown. Resource use, as in Table 1, is shown for each method.

**Supplementary Table 2**: Twenty gene trees most distant from the core genome tree in 616 *Streptococcus pneumoniae* genomes when using the KC metric with *λ* = 1, which only considers branch lengths. The name of the gene, or its name in the *S. pneumoniae* ATCC 700669 genome is shown with the annotated function. Whether each gene was found to be a recombination hotspot in the PMEN1 clone, and whether the hotspot has been specifically described previously are also shown.

**Supplementary Table 3**: Distance to the true tree for comparable models and methods. Three evolutionary models available both in IQ-tree and SEAVIEW, which were then used to build phylogenies using maximum likelihood (ML) or distances (BIONJ) respectively. Each model has an increasing number of degrees of freedom (df). The KC distances for topology ( *λ* = 0) and branch length ( *λ* = 1) are shown, along with the CPU time used for ML inference.

**Supplementary Figure 1**: An overview of the simulation procedure. Blue boxes show input data: a starting tree and genome at the root, for both evolutionary simulators ALF and DAWG; parameters for each simulator. Orange diamonds show processes: the simulators ALF (for genes) and DAWG (for intergenic regions); perl scripts to combine these results maintaining changes in gene order; pIRS to simulate error-prone reads. Yellow boxes show simulation output data: the full genomes for each sample at the tips of the input tree; aligned sequences for each gene; error-prone reads from the genomes.

**Supplementary Figure 2**: Applying *plotTreeDiff* between true tree and the closest reconstruction, RAxML + 23F aln (distance 4.35). See top an for explanation of *plotTreeDiff*.

**Supplementary Figure 3**: Applying *plotTreeDiff* between true tree and one a little further away, the fast IQ-tree (distance 11.3). See top for an explanation of *plotTreeDiff*.

**Supplementary Figure 4**: Applying *plotTreeDiff* between the true BIGSdb-like (distance 149.8). See top for an explanation of *plotTreeDiff*.

**Supplementary Figure 5**: Applying *plotTreeDiff* between the true and furthest, UPGMA + NCD (distance 210.5). See top for an explanation of *plotTreeDiff*.

**Supplementary Figure 6**: A multi-dimensional scaling plot of the distances between all methods projected into two dimensions. This view is zoomed, so the worst methods are outside the plot boundaries.

**Supplementary Figure 7**: A multi-dimensional scaling plot of the distances between trees sampled from the posterior using mrbayes, projected into two dimensions. There are two chains with different starting points, and the true tree is shown. Both chains appear to have converged on the same regions of treespace (no clustering by colour). There are two favourable modes in this topology space, one of which is closer to the true tree, but less frequently sampled than the other.
