# Supplementary material for: Evaluation of phylogenetic reconstruction methods using bacterial whole genomes: a simulation based study
Source: Wellcome Open Res. 2018 May 29;3:33. Originally published 2018 Mar 23. [Version 2] doi: 10.12688/wellcomeopenres.14265.2 (PMC5930550; doi:10.12688/wellcomeopenres.14265.2)
Supplement: Supplementary file 1 [file wellcomeopenres-3-15939-s0000.tgz › fa0c995b-0b41-4fa4-8c83-6eb71d62ced9.pdf]

Supplementary tables

Supplementary table 1: Comparison of phylogeny accuracy using all positions versus SNPs plus an ascertainment bias correction for maximum likelihood methods. The KC distance from the true tree, using topology only ($\lambda = 0$) and including branch lengths ($\lambda = 1$) is shown. Resource use, as in table 1, is shown for each method.

Method	Sites used	KC distance $\lambda = 0$	$\lambda = 1$	CPU time	Max memory
IQ-TREE	All	11.31	1.57	11hr 43min	3.2Gb
	SNPs + ASC	11.53	0.60	2hr 45min	0.55Gb
RAxML	All	11.31	1.57	9hr 47min	3.0Gb
	SNPs + ASC	11.53	0.89	1hr 54min	0.50Gb

Supplementary table 2: Twenty gene trees most distant from the core genome tree in 616 *S. pneumoniae* genomes when using the KC metric with $\lambda = 1$, which only considers branch lengths. The name of the gene, or its name in the *S. pneumoniae* ATCC 700669 genome is shown with the annotated function. Whether each gene was found to be a recombination hotspot in the PMEN1 clone, and whether the hotspot has been specifically described previously are also shown.

Gene	KC distance	Function	Recombination	Known hotspot
nanA	80.3	neuraminidase	yes	self
yvdC	76.8	CAAX amino terminal protease	yes	adjacent to *folA*/*dyr*
rafF/*msmF*	74.0	multiple sugar-binding transport system permease protein	yes	no
rafG/*msmG*	72.8	multiple sugar-binding transport system permease protein	yes	no
ftsL	61.8	Cell division protein	yes	adjacent to *pbp2x*/capsule
rplR	55.3	50S ribosomal protein L18	no	NA
FM211187.611	52.0	GNAT acetyltransferase	no	NA
aliB	49.7	Oligopeptide ABC transporter solute-binding protein	yes	capsule
artP	49.3	Arginine ABC transporter permease	yes	no
rsmC	48.7	16S rRNA methylase	no	NA
yvfR	45.4	ABC exporter ATPase	no	NA
mraY	42.6	Phospho-N-acetylmuramoyl-pentapeptide-transferase	yes	adjacent to *pbp2x*/capsule
queF	41.3	NADPH-dependent 7-cyano-7-deazaguanine reductase	yes	*psrP*
phoB	41.1	Response regulator	yes	*cbpA*
pbp1a	39.5	penicillin binding protein	yes	self
FM211187.4250	39.4	membrane protein	no	NA
lacE2	39.3	lactose-specific phosphotransferase system (PTS)	no	NA
dacB	35.5	D-alanyl-D-alanine carboxypeptidase	no	NA
ygaZ	33.9	Branched-chain amino acid AzlC-type transporter permease	yes	prophage insertion site
folP	33.6	dihydropteroate synthase	yes	adjacent to *folA*/*dyr*

Supplementary table 3: Distance to the true tree for comparable models and methods. Three evolutionary models available both in IQ-tree and SEAVIEW, which were then used to build phylogenies using maximum likelihood (ML) or distances (BIONJ) respectively. Each model has an increasing number of degrees of freedom (df). The KC distances for topology ($\lambda = 0$) and branch length ($\lambda = 1$) are shown, along with the CPU time used for ML inference.

Evolutionary model	df	Method	KC distance		CPU time
			$\lambda = 0$	$\lambda = 1$	
JC/JC69	0	BIONJ	158.0	1.69	-
		ML	11.3	1.59	3hr 48min
K2P/K80	1	BIONJ	119.3	5.12	-
		ML	11.3	1.59	4hr 45min
HKY/HKY85	4	BIONJ	158.0	1.69	-
		ML	158.0	1.59	5hr 46min

Supplementary figures

These are static figures, which can also be viewed as html at `https://dx.doi.org/10.6084/m9.figshare.5923300`. Supplementary figs. 2 to 5 show *plotTreeDiff* results between the true tree and a reconstruction. These shows tips with topology differences between the two trees: the ancestors of grey tips in the first tree have the same partition of tip descendants in the second tree; tips are otherwise coloured by the number of mismatches amongst its most recent common ancestors with other tips. Red tips have the most ancestral differences, and blue the least.

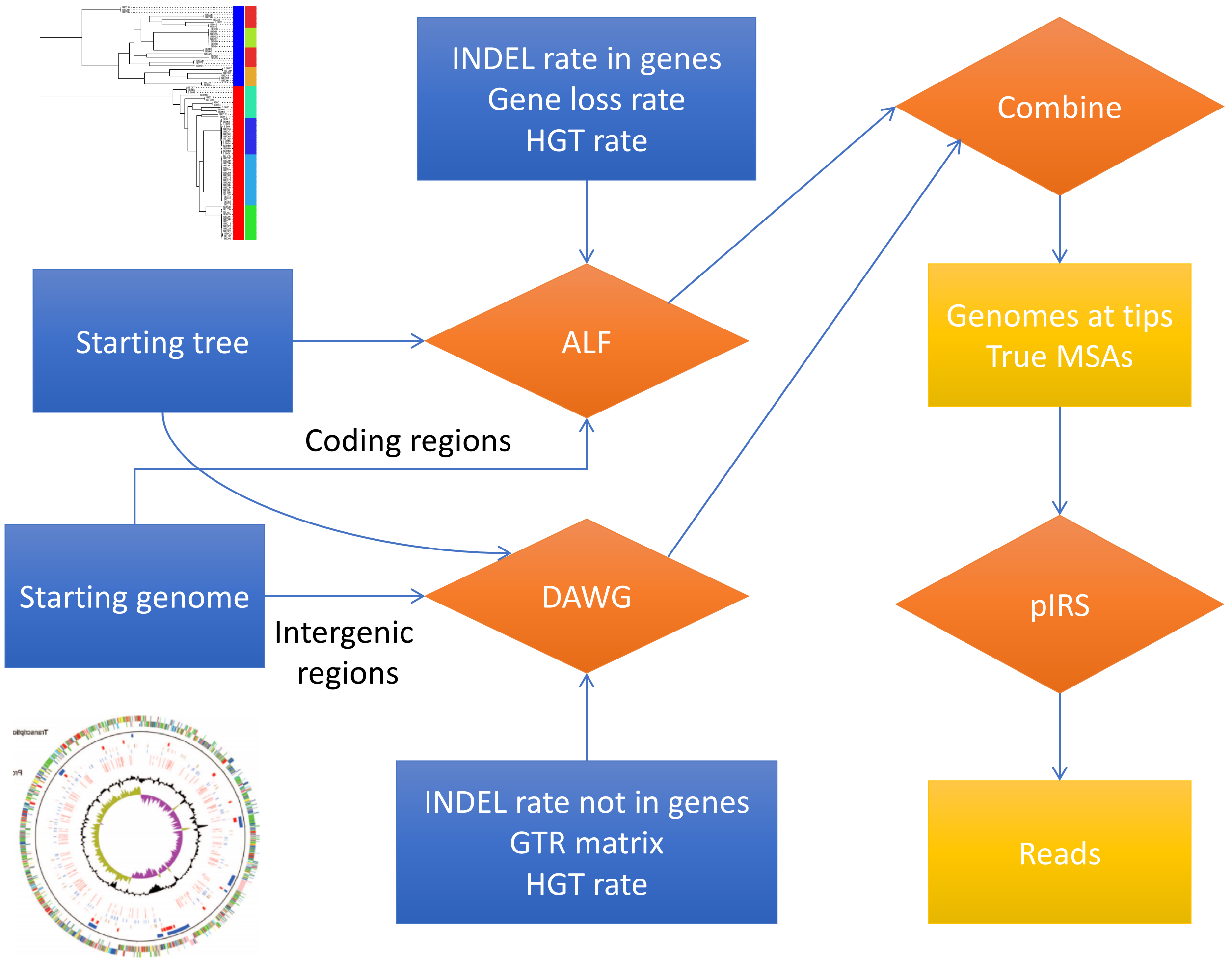

Supplementary figure 1: An overview of the simulation procedure. Blue boxes show input data: a starting tree and genome at the root, for both evolutionary simulators ALF and DAWG; parameters for each simulator. Orange diamonds show processes: the simulators ALF (for genes) and DAWG (for intergenic regions); perl scripts to combine these results maintaining changes in gene order; pIRS to simulate error-prone reads. Yellow boxes show simulation output data: the full genomes for each sample at the tips of the input tree; aligned sequences for each gene; error-prone reads from the genomes.

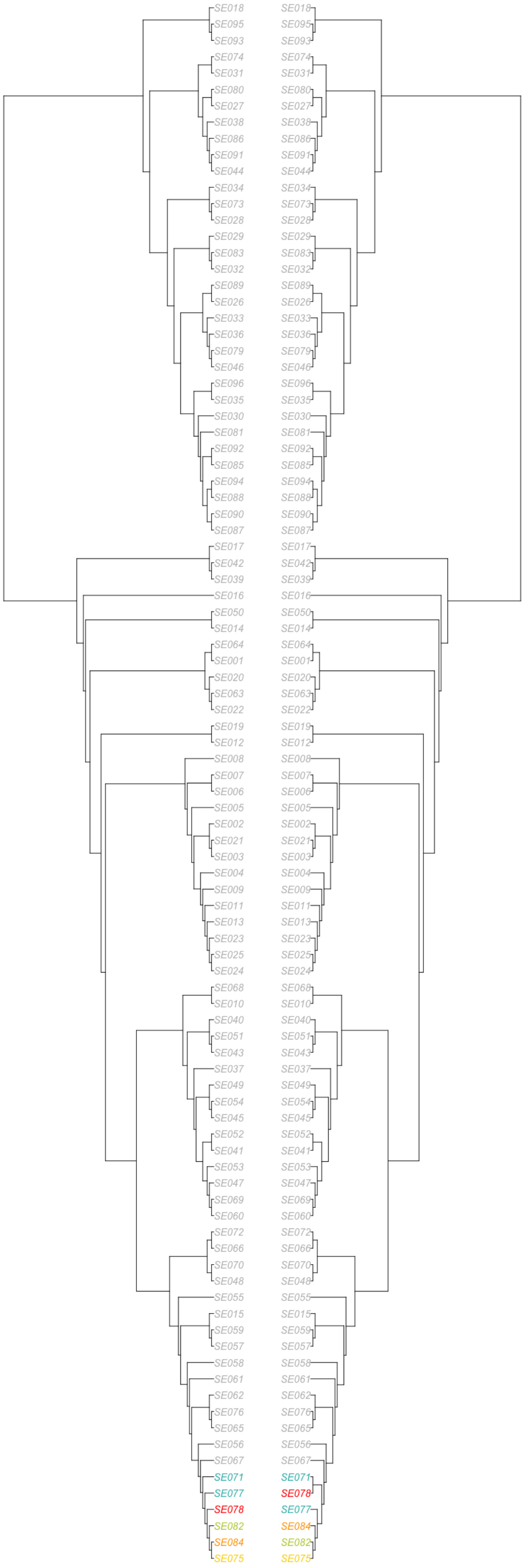

Supplementary figure 2: Applying *plotTreeDiff* between true tree and the closest reconstruction, RAxML + 23F aln (distance 4.35). See top an for explanation of *plotTreeDiff*.

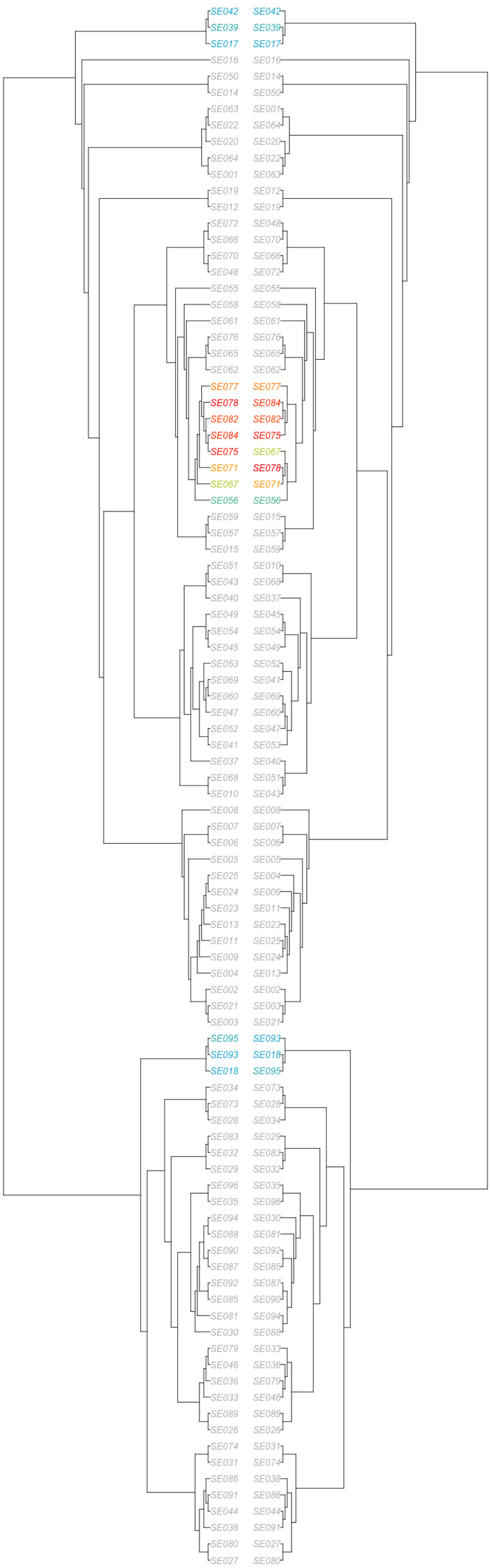

Supplementary figure 3: Applying *plotTreeDiff* between true tree and one a little further away, the fast IQ-tree (distance 11.3). See top for an explanation of *plotTreeDiff*.

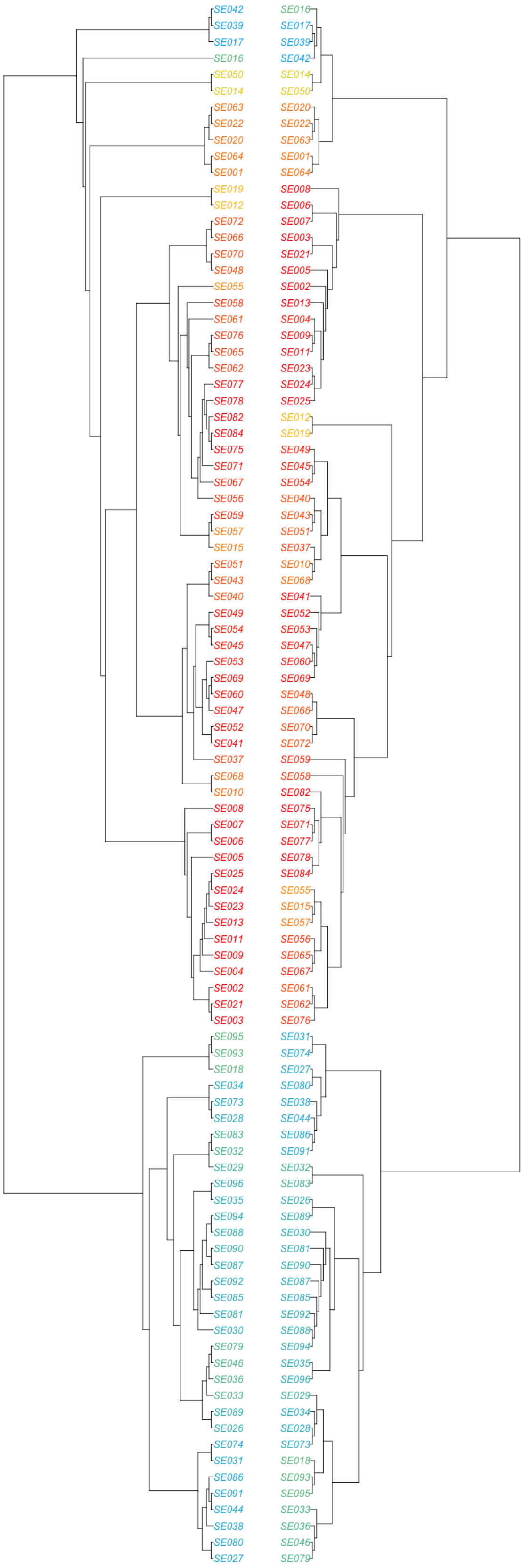

Supplementary figure 4: Applying *plotTreeDiff* between the true BIGSdb-like (distance 149.8). See top for an explanation of *plotTreeDiff*.

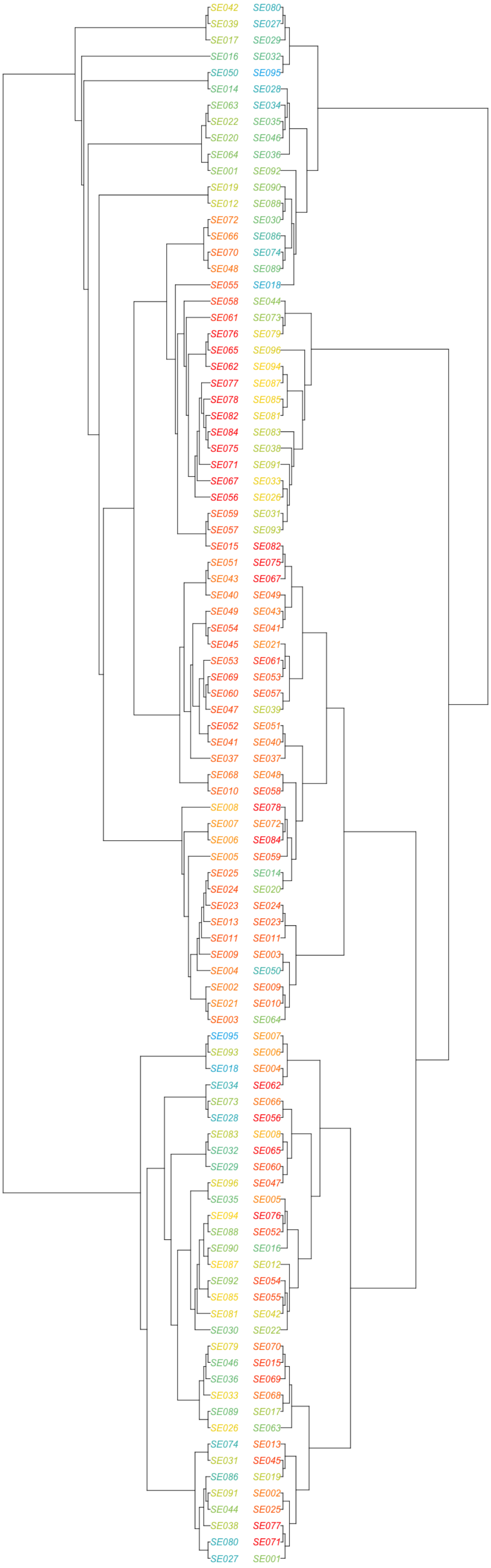

Supplementary figure 5: Applying *plotTreeDiff* between the true and furthest, UPGMA + NCD (distance 210.5). See top for an explanation of *plotTreeDiff*.

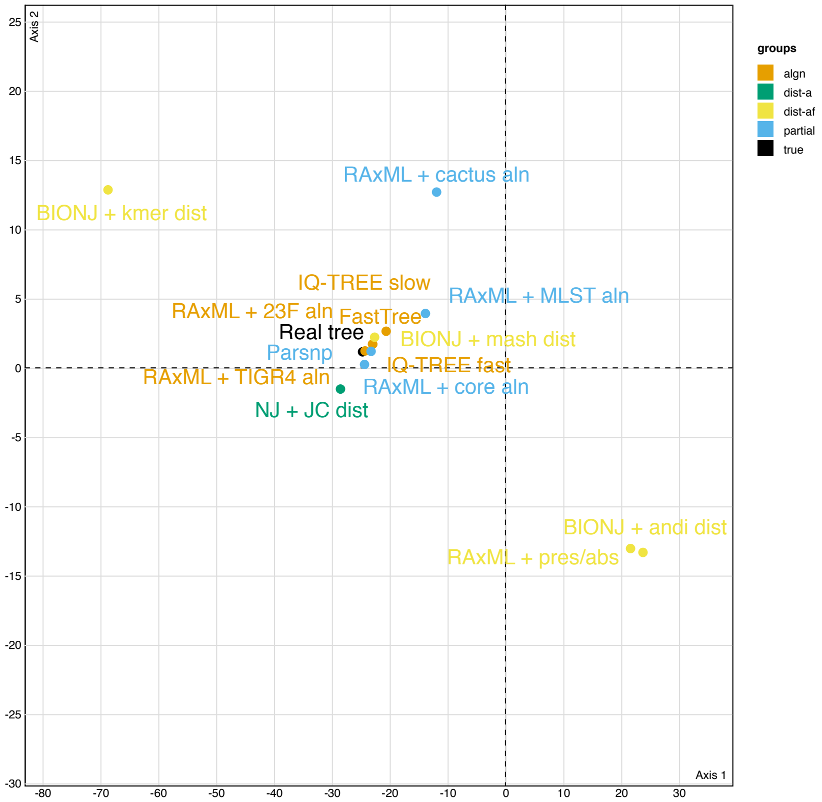

Supplementary figure 6: A multi-dimensional scaling plot of the distances between all methods projected into two dimensions. This view is zoomed, so the worst methods are outside the plot boundaries.

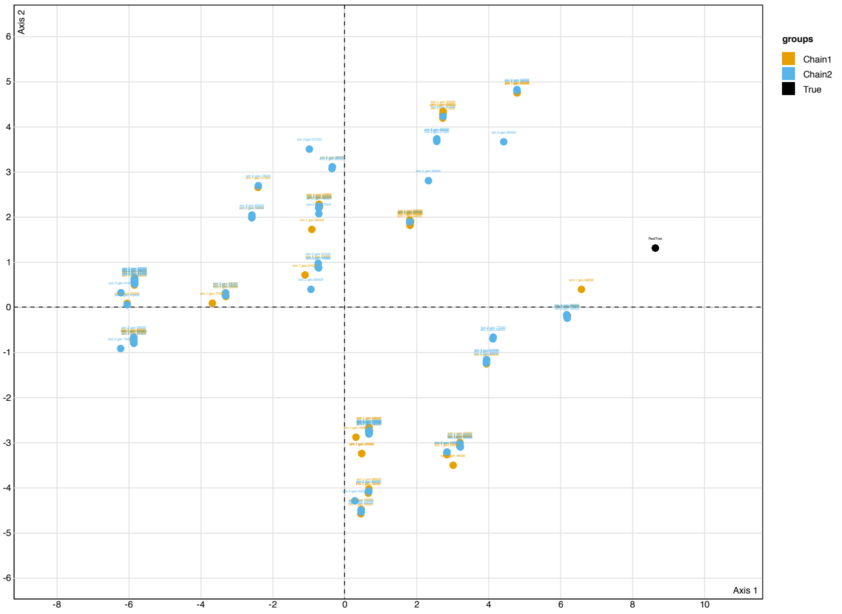

Supplementary figure 7: A multi-dimensional scaling plot of the distances between trees sampled from the posterior using mrbayes, projected into two dimensions. There are two chains with different starting points, and the true tree is shown. Both chains have converged (no clustering by colour). There are two favourable modes in this topology space, one of which is closer to the true tree, but less frequently sampled than the other.
