# Supplementary material for: Evaluation of phylogenetic reconstruction methods using bacterial whole genomes: a simulation based study
Source: Wellcome Open Res. 2018 May 29;3:33. Originally published 2018 Mar 23. [Version 2] doi: 10.12688/wellcomeopenres.14265.2 (PMC5930550; doi:10.12688/wellcomeopenres.14265.2)
Supplement: Peer review history

# Peer review: Referee response for version 2

**DOI:** 10.21956/wellcomeopenres.15939.r33217

**Published:** 2018-05-30

**Authors:** Lauren A. Cowley, Taj Azarian

**Affiliations:** 1 Harvard T.H. Chan School of Public Health, Boston, MA, USA; 2 Center for Communicable Disease Dynamics, Department of Epidemiology, Harvard University, Boston, MA, USA

Thank you very much to the authors for addressing all our points and updating the manuscript accordingly, we think the manuscript reads extremely well now.

We have read this submission. We believe that we have an appropriate level of expertise to confirm that it is of an acceptable scientific standard.

# Peer review: Referee response for version 1

**DOI:** 10.21956/wellcomeopenres.15526.r32392

**Published:** 2018-04-30

**Authors:** João A. Carriço

**Affiliations:** 1 Faculty of Medicine, Institute of Molecular Medicine, University of Lisbon, Lisbon, Portugal

The article by Lees et al. presents us with a very needed evaluation of currently phylogenetic reconstructions methods, based on a simulation based approach. It is a very well written article in a much-needed area and provides several important messages to researchers in this field. I thank the opportunity to review such interesting and important work.

 There are however some points that I believe would help the readers in better understanding the details of the analysis, and some further information could help the study reproducibility and replication of results (these are the points that I reported as Partly on my report) and my questions will focus on them.

 I will provide the comments per section:

**Introduction**

 Very well written, succinct and full of important and relevant references.

1. (Last sentence of introduction) Concerning providing the code and simulated data, I think all the figshare files and github repositories are in need of a readme file which should contain a better description of the commands and parameters used with some examples to reproduce the paper. Otherwise the claim of reproducibility cannot be made. Even consider a repository for the simulated reads used in this study.

 

**Methods** 

 The methods used by the authors show a tremendous amount of work using several software available to reach their goals. This is highly commendable, but unfortunately also implies that partial description of each software is needed to follow-up without the need of re-reading all the original articles. My following comments are done having this in mind, and to facilitate the reproducibility of the steps:

1. The authors state that they used ALF 1.0 to simulate the evolution along a given phylogenetic tree of 2232 CDS of S. pneumoniae ATCC 700669. I assume that ALF must have some stochastic step and, if such, a seed should be provided to reproduce the same results. Furthermore the phylogeny used was a from a core alignment of Listeria monocytogenes that also has a BAPS classification. At first sight this can be rather confusing for the reader. If I understood correctly, It should be clarified that from the starting CDS, ALF was used to create a final tree with 96 simulated S.pneumoniae strains from the original ATCC 700669, that would correspond to the same topology as the tree from Kremer et al. I also assume that the BAPS groups were recalculated from the final genomes. If so it should be stated on the article.
2. The estimation of the rates to use in GTR the authors used 3 strains (2 pneumo and 1 mitis as outgroup. The claim that this “allowed calculation of SNP and INDEL rates across recent  S.pneumoniae evolutionary history” is a bit too extreme and should be moderated.
3. The authors then refer that used DAWG 1.2. to simulate evolution of inter-genic regions. Please clarify how these were defined. The initial text seemed only to refer to the 2232 CDS. Maybe this should be rephrased saying that both CDS and intergenic regions of ATCC 700669 were used in simulating the evolution. Furthermore, the authors should explain how these two approaches can be reconciled in a unique analysis, or at least explicitly state the artificial nature of the result (which I don’t believe that has any impact for the purpose of the paper but should be clarified)
4. Why the choice of velvet for the assembler? Spades has been shown to provide much better results. Furthermore, what were the parameters for velvet? Consider providing the command lines (as supplemental material) for de novo assembly by velvet, for bwa-mem, samtools and roary, as it will be very useful for readers that are new to the field.
5. Consider presenting a summary figure of the whole simulation process, since it would help to guide the reader through the multiple steps done.
6. MLST: why choose 7-genes at random and not use the ones from the schema? I believe that this can have highly misleading results when compared with the defined MLST schema and defining this as MLST analysis mislead the readers.
7. Methods of phylogeny reconstruction and Table 1. Consider numbering the enumeration of methods presented in the text and make a correspondence in a column in Table 1. As it is it is not easy to make the correspondence. For BIGSdb, how was missing data handled and what core schema was used?
8. The Quantification of differences between phylogenetic tree topologies using the KC metric was an excellent choice and the supplemental figures 1 to 3 are really illustrative examples. How are the randomly generated trees generated? This should be added to this section.
9. Core gene trees from real data. The use of MDS to visualize the pair-wise distances is really necessary? An ordered heat-map of the KC metric for the samples would give similar information? I understand the use of the MDS but my feeling is that the final comparison can be biased by the methodology.

 

**Results**

1. “We note that all methods except for the NCD were able to recapitulate the population clusters as defined by BAPS” I think this is an important conclusion because in many applications of the trees, researchers compare partitions of the tree and not topology to arrive to their conclusions. In my opinion this should be revisited in the Discussion.  
2. Table 1: Add to the table legend the meaning of the “Accessory Genome”  column. Also clarify in the text what is the meaning of BAPS 1 and 2.  Also explain the meaning of “all” (UPGMA+NCD) in the legend)
3. “However, there is the issue that when a variant is present in a region overlapped by two genes it will be erroneously represented twice.” What is a region overlapped by two genes in this context? Were the CDS defined to allow this? This also raises the question what was considered CDS ? Was it what was defined in the previous annotation?  
4. Figure 3. See my previous comment to the use of MDS. Also the core genome tree does not behave as average (or centroid) in this dataset and as appears it seems biased to the left of the clusters. I believe that this can be a by-product of the MDS dimensionality reduction. A very interesting result, is what concerns the ribosomal genes. This seems to clearly point out that their use is bad in recapitulating phylogeny and I wonder of this is not only due to the artificial nature of the dataset and similar studies in other species and other might elucidate this matter. It would be interesting to reconcile such results with the results obtained from ribosomal MLST for example in real datasets.
5. “When picking an MLST scheme for an organism, given a choice of genes to use, these phylogenetic signals may be a useful additional consideration.” This sentence could be better explained, since it seems really relevant. Could this approach be used as a method to evaluate the choice of MLST target loci for each species?
6. “Although O( *N* ^2^) distances need to be evaluated” – You mean *N* ^2^ distances. No need for O notation here.  
7. On BIGSdb “However, this approach also limits resolution and inference of intra-cluster distances, and produced one of the worst topologies in our tests.” Where were the topologies mismatches more common? Within each cluster? Or between clusters? This is relevant because the way information of allelic profiles is commonly used.

 

**Discussion**

 Well written and informative. The caveats of this study are presented in a paragraph. I think the results of this simulation provide good insights but I wouldn’t extrapolate to any other species and dataset. Monomorphic and fastidious species would probably have more similar results using any approach and a study on the impact of mutation and recombination parameters on the final tree-of-trees would be very interesting to see as a future follow-up study.

I have read this submission. I believe that I have an appropriate level of expertise to confirm that it is of an acceptable scientific standard, however I have significant reservations, as outlined above.

# Peer review: Referee response for version 1

**DOI:** 10.21956/wellcomeopenres.15526.r32389

**Published:** 2018-04-20

**Authors:** Philip M. Ashton

**Affiliations:** 1 Oxford University Clinical Research Unit, Ho Chi Minh City, Vietnam

The manuscript from Lees and colleagues aims to describe the accuracy and speed of a wide variety of methods for the construction of phylogenetic trees. They achieve this aim in a generally readable and very informative paper. They show that maximum likelihood based approaches using alignment to a closely related reference genome provide the best inference of the simulated true phylogeny. There are various other interesting nuggets spread throughout the paper and it is an interesting read for anyone working with bacterial phylogenies.

 I was also interested to note the authors decision to submit to Wellcome Open Research. My hope is that they will take advantage of Wellcome Open Research allowing updating of articles with 'minor' new analyses to include new software which may be released for phylogenetic analysis.

 The article is a nice crystallisation and examination of many pieces of received wisdom in bacterial phylogenomics community, especially the balance between accuracy and speed for mash/kmer trees, NJ trees of alignment data and ML trees of alignment data.

 I think the work is well presented, well carried out and the conclusions do not over-reach the results.

**Minor comments**

- In the introduction, this sentence doesn't make sense - 'A more recent, larger study in eukaryotes compared these an IQ-TREE in terms of best likelihood on both species and gene trees'
- As the authors and other reviewers allude to, it is sometimes forgotten that a single tree is not a very realistic representation of the output of an ML phylogenetic analysis. It would be interesting to try and represent this somehow for the different methods. Perhaps a visualisation along the lines of supp figure 4, but with 100 bootstraped trees per method, or the 100 trees with the best ML scores. I appreciate that this is already a busy figure, so I leave it up to the authors whether to do this, or if there is a better way to do it.
- The authors have uploaded scripts to an accompanying github repo, but there is no readme or guide to which scripts relate to which parts of the paper. This should be improved.

I have read this submission. I believe that I have an appropriate level of expertise to confirm that it is of an acceptable scientific standard.

# Peer review: Referee response for version 1

**DOI:** 10.21956/wellcomeopenres.15526.r32391

**Published:** 2018-04-09

**Authors:** Lauren A. Cowley, Taj Azarian

**Affiliations:** 1 Harvard T.H. Chan School of Public Health, Boston, MA, USA; 2 Center for Communicable Disease Dynamics, Department of Epidemiology, Harvard University, Boston, MA, USA

**We have decided to provide a joint review from two postdocs in the Hanage lab of “Evaluation of phylogenetic reconstruction methods using bacterial whole genomes: a simulation based study”.**

**Lauren Cowley’s review:**

 I am grateful for the opportunity to review “Evaluation of phylogenetic reconstruction methods using bacterial whole genomes: a simulation based study” by Lees et al. I found it very interesting and especially useful and relevant to my own research. I am sure I will refer back to it a few times in the future. I also think it will be highly valuable to the wider community of researchers doing bacterial genomic research, which is growing. I think it is likely to become a highly cited paper.

 The authors provide a thorough and well thought out analysis of different methods for inferring phylogeny from bacterial datasets. They have also provided details of the computational time and memory required for each method. They have represented their work clearly and produced very informative figures that are extremely useful to the reader. I recommend this paper for publication and just have a few thoughts:

**Positive points:**

- Very interesting paper
- It’s a very useful paper for picking the most appropriate method
- The figures are very nicely produced
- Figure 4 is fantastic.

**Major revisions:**

1. Would be nice to see this kind of analysis for rooting affects. Could you do alignments with and without an outgroup to show which methods were close to the right rooting/ordering without the outgroup?
2. Another distance matrix software that you could include is called Disty McMatrixface, would be nice to see if there is any variation in that?
3. You state you selected the MLST genes at random, were the genes not checked for being under selective pressure or likely to recombine? MLST genes are not chosen at random and are usually housekeeping genes that are not expected to recombine a lot or be under particular selective pressures. This will affect that aspect of the analysis.
4. It is very intuitive that the genes with discordant trees are recombination hotspots, none of the analysis was run with a post gubbins alignment? Would it not be important to include this?

**Minor revisions:**

1. You generated error prone illumina reads with pIRS, is there any variation from wgsim?
2. I’m interested that you used velvet instead of SPAdes? I have usually found SPAdes better for bacterial assembly. What assembly parameters did you use? What K size? You say you improved the resulting scaffolds? How? Assembly quality will greatly affect the Parsnp analysis, there should be some mention of that.
3. You state that including the accessory genome is ok in Pneumo but it would be nice to state that for E. coli this is very inadvisable, maybe also give some other examples where the accessory genome would affect this kind of analysis.
4. I like table 1, I think an extra column with recommended use would be helpful. You mention a few times in the text where you might choose that form of analysis but it would be nice to summarise it in the table too.

 

**Taj Azarian’s review:**

 Thank you for the opportunity to review “Evaluation of phylogenetic reconstruction methods using bacterial whole genomes: a simulation based study” by Lees and colleagues. In their analysis, the authors methodically assess a variety of methods to infer a phylogeny from a bacterial genomic dataset. They compare the ability to recover a “true tree” as well of the computational time required using different phylogenetic reconstruction methods. I feel this is much needed work, as we seem to have drifted away from the phylogenetic tree as the central finding, now just an intermediate step in analysis of large bacterial genomic datasets. As such, many often overlook the implication of their alignment and phylogeny inference methods. Overall, the manuscript is consummate. I include some general comments and suggestions below, which I feel would strengthen their manuscript. 

1. There should be some focused text in the introduction or discussion about the users goal of phylogenetic reconstruction and how this would possibly determine the analytical approach (i.e., why are you making a tree?). If population structure is your main goal, then almost all approaches will recover the correct level 1 and 2 BAPS clusters. If you are more interested in investigating transmission or the association of epidemiological traits, then perhaps the resolution in tip branch-lengths and topology is important; therefore, an approach that uses a reference-based alignment may be better. It could also be clarified that use of a core-genome alignment at the species level (i.e., not just a lineage/clone) could result in a good amount of signal loss within the BAPS clusters (which is why a lot of these trees have pancaked clades). Further, if more resolution is desired, then reference-based alignment may be performed on a specific BAPS cluster using a close reference (something that is often done in practice). Last, it may be worth including a reminder that violations in some if not all assumptions are made when inferring a phylogeny from a bacterial dataset. As you state, the “true tree” is almost never recovered, but I feel like a lot of researchers forget there are assumptions that are made every time you infer a tree. Certainly, not all of the above needs to be included, but some consideration should be made to incorporating these concepts into the text.
2. It should be stated up front the reason for only comparing tree topology and not branch lengths. I am assuming this was done because branch lengths using distance and character-based tree inference methods would vary, possibly unfairly biasing toward ML trees. In addition, the change in number of sites used would affect branch lengths (core vs reference vs MLST), and none would necessarily be “wrong”.
3. Have the authors explored how the true tree topology (regarding the “qualities” they mention) may impact the performance of various phylogeny inference approaches? For example, it is known that UPGMA perform particularly bad in certain situations. The authors mention this in the discussion regarding varying degrees of phylogenetic signal. I would imagine that with low signal, character based methods would perform better than distance-based approaches. Does this matter, or are the errors “washed-out” when using genome-wide data as seen with putative recently admixed genes?
4. Everyone has their own “pet” approach and the authors could spend a lifetime testing different combinations of methods. Having said that, there is one approach that I believe should be evaluated for its possible computational savings. I almost always use RAxML pthreads on SNP alignments using ascertainment bias correction because I have experienced (anecdotally) faster run times than using the full alignment. My understanding is that using only variant sites will impact branch lengths to some degree (because invariant sites are used in the likelihood calculation) but not the overall tree topology. I think it is worth trying and including if there are significant computational savings to using the full alignment. I would suggest the following: using either the core gene alignment or reference-based alignment, extract variant sites using SNP-sites. Then run RAxML something like this: raxmlHPC-PTHREADS-SSE3 -T 16 -f a -p 12345 -s alignment.fasta -x 12345 -# 100 -m ASC_GTRGAMMA -n alignment --asc-corr=lewis (note-1 this is for v8.2.1 which may be different for 7.8.6. note-2: you can remove the bootstrap option). See if there are memory and CPU time savings and then compare the topology. 

 

**Minor comments**

- Consider revising the conclusions in the abstract to include the best method for recovering the True Tree (RAxML + reference-based alignment)
- State whether Roary was used with the default PRANK codon aware alignment or mafft alignment.  PRANK takes considerably longer (as I am sure you know) and may only perform marginally better in terms of recovering branch lengths.
- In the methods, you mention that ,“Hamming distance between rows of the gene presence/absence matrix produced by Roary (using 95% blast ID cutoff).” Did you use the gene presence/absence output from Roary (accessory_binary_genes.fa only contains a subsample of all accessory COGs) or the entire accessory genome manually extracted from the gene_presence_absence.csv? If the prior, I would consider repeating using the entire presence/absence alignment.
- There are a few sentences that are a little hard to track due to length. For example, in the Methods on page 3, the sentence describing the test tree could be revised as follows: “We identified a phylogeny (Figure 1), originally produced by Kremer et al. from a core genome alignment of 96 *Listeria monocytogenes* genomes from patients with bacterial meningitis, which had a number of qualities we wished to be able to reproduce. Particularly, it possessed two distinct lineages (also making midpoint rooting suitable, and negating the strong dependence on correct rooting implicit in the Kendall and Colijn metric), several clonal groups within each lineage, long branches and a polyphyletic population cluster (population clusters were estimated from a core genome alignment using Bayesian Analysis of Population Structure v6.0 (BAPS)). “
- Another distance approach worth considering would be Torsten Seemens SNP-Dist https://github.com/tseemann/snp-dists since it is rapid and allows for raw SNP distances and simple models (JC, HKY etc).
- The authors state, “a possibility for combining these two data types would be to have separate model partitions for SNP variation and gene gain/loss.” This would indeed be very interesting.
- If the authors do consider SNP sites only, I would be interested in how the inclusion of gapped-sites of Ns impacts the results. 

We have read this submission. We believe that we have an appropriate level of expertise to confirm that it is of an acceptable scientific standard, however we have significant reservations, as outlined above.
